# Effect of Online Health Information Seeking on Anxiety in Hospitalized Pregnant Women: Cohort Study

**DOI:** 10.2196/16793

**Published:** 2020-05-06

**Authors:** Fabiana Coglianese, Giulia Beltrame Vriz, Nicola Soriani, Gianluca Niccolò Piras, Rosanna Irene Comoretto, Laura Clemente, Jessica Fasan, Lucia Cristiano, Valentina Schiavinato, Valter Adamo, Diego Marchesoni, Dario Gregori

**Affiliations:** 1 Unit of Obstetrics and Gynecology Maternal-Infant Department Santa Maria degli Angeli Hospital Pordenone Italy; 2 Department of Obstetrics Burlo Garofolo Pediatric Institute Trieste Italy; 3 Department of Cardiac, Thoracic, Vascular Sciences and Public Health University of Padua Padua Italy; 4 Department of Philosophy, Sociology, Education and Applied Psychology University of Padua Padua Italy; 5 Unit of Obstetrics and Gynecology Maternal-Infant Department Santa Maria della Misericordia Hospital Udine Italy

**Keywords:** anxiety, pregnant women, web health information, internet use

## Abstract

**Background:**

There are approximately 1,000,000 pregnant women at high risk for obstetric complications per year, more than half of whom require hospitalization.

**Objective:**

The aim of this study was to determine the relation between online health information seeking and anxiety levels in a sample of hospitalized woman with pregnancy-related complications.

**Methods:**

A sample of 105 pregnant women hospitalized in northern Italy, all with an obstetric complication diagnosis, completed different questionnaires: Use of Internet Health-information (UIH) questionnaire about use of the internet, EuroQOL 5 dimensions (EQ-5D) questionnaire on quality of life, State-Trait Anxiety Inventory (STAI) questionnaire measuring general anxiety levels, and a questionnaire about critical events occurring during hospitalization.

**Results:**

Overall, 98/105 (93.3%) of the women used the internet at home to obtain nonspecific information about health in general and 95/105 (90.5%) of the women used the internet to specifically search for information related to their obstetric disease. Online health information-seeking behavior substantially decreased the self-reported anxiety levels (*P*=.008).

**Conclusions:**

Web browsing for health information was associated with anxiety reduction, suggesting that the internet can be a useful instrument in supporting professional intervention to control and possibly reduce discomfort and anxiety for women during complicated pregnancies.

## Introduction

Approximately 1,000,000 pregnant women are at high risk for obstetric complications globally per year, about 700,000 of whom will require hospitalization. Preterm labor, placenta previa, pregnancy-induced hypertension, and gestational diabetes are some of the most common conditions during pregnancy that require medical attention [1,2].

Moreover, mental disorders can affect the pregnancy course, especially in high-risk pregnancies, which can exacerbate depression and anxiety, and hospitalization can further increase stress levels [3,4]. According to the World Health Organization, mental health disorders are the leading cause of disease burden in women aged between 15 and 44 years [5], corresponding to the main fertile window. In this regard, depression or anxiety during pregnancy has been associated with poor maternal health behaviors (eg, tobacco use) and with adverse birth outcomes (eg, preterm labor). Moreover, anxiety or depression during pregnancy may also adversely affect the development of the infant/child [6-8].

Antenatal depression and anxiety occur in approximately 13% and 21.7% of women, respectively [9]. The former affects 19% of women hospitalized for obstetric risk [3], whereas about 1 out of 3 pregnant women suffers from anxiety. In particular, the prevalence of depression and anxiety is higher in the first and third trimester (36.3% and 35.8%, respectively) and is slightly lower during the second trimester (32.3%) [3,10].

Despite the relevance for pregnancy outcome, mental health of the mother, and development of the child, few studies have directly explored depression, anxiety, quality of life, and possibilities of mental health treatment in women hospitalized for high-risk pregnancies. The coexistence of anxiety and depression in this vulnerable group reaches up to 40%, which is 3 times greater than the rate reported in community-based samples of pregnant women [10].

Online health information-seeking behavior has become increasingly popular among pregnant women owing to the several uncertainties that can arise during pregnancy [11-14]. Moreover, health care professionals often provide pregnant women with informational support, especially underlining where and how to obtain the resources they need [15,16]. Although many studies have investigated the psychological and environmental factors predisposing subjects to online health information-seeking behaviors, only few have examined the effect of this behavior and its relationship with anxiety [16].

Therefore, the aim of this study was to determine the relationship between online health information-seeking behaviors and anxiety levels among a sample of women hospitalized for a pregnancy-related issue. Moreover, we aimed to understand how anxiety levels change during hospitalization by comparing anxiety levels and access to online health information between women having access to the internet during hospitalization and those who did not.

## Methods

### Study Design and Setting

We performed a two-center cohort study at the Departments of Obstetrics of Santa Maria della Misericordia Hospital in Udine and Santa Maria degli Angeli Hospital in Pordenone, Italy between August 2015 and March 2016. Women enrolled in the study were >18 years old and 1-40 weeks pregnant who were hospitalized for obstetrics-related complications, including gestational diabetes, preeclampsia, pregnancy-induced hypertension, renal colic, and severe hyperemesis. Women without pathological pregnancy, with cognitive or major psychiatric diseases, and nonnative speakers of Italian were excluded from the study. To minimize bias, both nursing and midwifery staff were instructed not to interfere with patients’ spontaneous usage of internet resources.

Data collection was based on electronic case report forms maintained on the Research Electronic Data Capture [17] system of the Service for Clinical Trials and Biometrics of the Unit of Biostatistics, Epidemiology and Public Health (Department of Cardiac, Thoracic, Vascular Sciences and Public Health, University of Padova, Italy). The study received authorization by the Region Ethical Committee (CERU; protocol 17002, Opinion no. 37/2015, 7/7/2015). Informed consent was obtained from all individual participants included in the study.

### Data Collection

The pregnant women enrolled in the study were asked to fill out various questionnaires during hospitalization: Use of Internet Health-information (UIH) questionnaire about use of the internet, EuroQOL 5 dimensions (EQ-5D) questionnaire on quality of life, State-Trait Anxiety Inventory (STAI) questionnaire for measuring two distinct anxiety concepts, and a questionnaire about critical events occurring during hospitalization. Additional demographic and clinical information, including gender, age, education level, obstetric history (childbirth, miscarriage, ectopic pregnancy, and type of pregnancy problem), and use of alcohol and tobacco, were collected by the study researchers based on medical histories. Critical events such as medical complications, hospital dissatisfaction, and family problems occurring during the hospitalization period were also recorded.

### Internet Health Information Questionnaire

The UIH questionnaire on online information-seeking behaviors [18-20] was adapted for this study population. This questionnaire is divided into 3 parts. The first part investigates internet usage at home, patients’ attitude with respect to searching health information, the type of information most frequently searched for, the general frequency of web use, and the tendency to share this information with health care providers, usually a midwife. This part of the questionnaire was administered only at the beginning of hospitalization. The second part, which was administered every day until discharge, investigated internet usage during the hospital stay, the tool used (eg, smartphone, tablet, notebook), and the time spent searching for information about their health condition. The third part was composed of a visual analog scale (UIH-VAS) regarding the amount of time spent on the internet in the last unit of time (usually the day) in searching for information about a specific disease or regarding general health-related information.

### EuroQOL 5 Dimensions Health-Related Quality of Life Questionnaire

The EQ-5D questionnaire [21] was adopted to measure health-related quality of life, which consists of a questionnaire and a visual analogue scale (EQ-VAS). The EQ-VAS records subjects’ perceptions of their own current health status and can be used to monitor changes over time. The questionnaire is a self-reported description of subjects’ current health in five dimensions: mobility, self-care, usual activities, pain/discomfort, and anxiety/depression. Subjects are asked to grade their own current level of function in each dimension choosing between three degrees (severe, moderate, or none). Combining the different information, 245 distinct health states can be described.

### State-Trait Anxiety Inventory

The STAI [22], which was adapted for the Italian population [23], is a questionnaire frequently used in pregnant women affected by obstetric diseases to evaluate nonpathological anxiety levels. The STAI is composed of two self-reported scales for measuring two distinct anxiety concepts: state anxiety and trait anxiety. Both scales contain 20 statements that ask the respondents to describe how they feel at a given time (state anxiety) and how they generally feel (trait anxiety). In this way, state anxiety is conceptualized as a transitory emotional state, whereas trait anxiety refers to relatively stable individual differences in their propensity for anxiety. The state anxiety questionnaire was administered every day until discharge, whereas the trait anxiety questionnaire was filled out only once at the beginning of hospitalization.

### Sample Size Calculation

This research was powered to detect potential differences on the average STAI score (range 20-80) of 6 points between women with internet access (with a minimum of 10 minutes/day of web browsing, excluding emails) and those without internet access. Based on previous estimates using the same instrument [24], assuming an SD of 8 points in the differences of STAI scores and assuming a ratio of 0.42 between the rate of women with and without internet access (for α=.03 and 1 – β=.85), a total of 105 pregnant women were planned to be recruited (using a two-sample *t* test with unknown variance).

### Statistical Analysis

Descriptive data are presented as the median (IQR) for continuous variables and as absolute numbers (percentages) for categorical variables as appropriate. Unadjusted differences were tested using Wilcoxon or Chi square tests without continuity corrections as appropriate depending on the variable analyzed.

Effects of relevant confounders on STAI scores and internet usage were considered by estimates in a multivariate longitudinal linear model. The marginal effects of relevant covariates were estimated using the Huber-White sandwich estimator and an autoregressive correlation structure [25]. Variables were selected from a pool of significant variables based on univariate analyses according to an Akaike information criterion value at least 0.25 [26] in a forward fashion with a significance threshold of *P*=.10. Age and quality of life, measured by the EQ-VAS, were forced to stay in the model regardless of their significance. Nonlinear effects of covariates were estimated using restricted cubic splines and their significance was estimated using a log-likelihood ratio test. A specific term for the interaction between UIH and time was added to the final model to evaluate its statistical significance and was eventually removed if the corresponding *P* value fell below .05. Goodness of fit was evaluated using the *R^2^* value on a set of bootstrapped (B=10,000) resamples. The analysis was performed using the RMS libraries [27] and R software packages [28].

## Results

A total of 105 hospitalized pregnant women were recruited for the study. The main characteristics of the study sample are provided in Table 1, stratified by internet usage group. Overall, the preferred tool for internet use was a personal computer.

With respect to internet usage, 98 out of 105 women (93.3%) reported using the internet at home not only for emails but also to seek health-related information. The majority of women were looking for health or medical information for themselves (95/105, 90.5%) or for someone else (70/105, 66.7%); to request personal health information such as test results or medical appointments (72/105, 68.6%); to communicate with physicians (55/105, 52.4%); or to consult informational websites about weight, diet, or physical activity during pregnancy (36/105, 34.3%). Moreover, 95 of the 105 subjects (90.5%) used internet specifically to obtain information on their obstetric disease: 85 of 95 women (89%) found the information useful, whereas only 44 of 93 participants (47%) shared the information they found with their health care providers. Only 7 of the total 105 women (6.7%) had not been using the internet at home.

**Figure 1:**
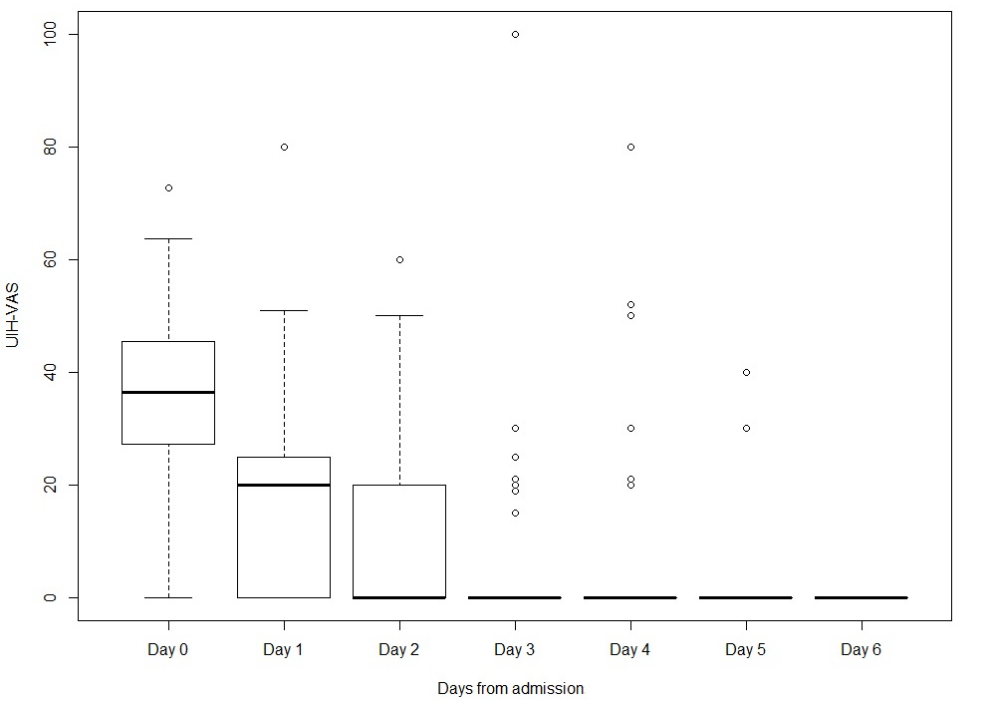
Use of Internet Health-information Questionnaire (UIH)-visual analog scale (VIS) levels over the days spent in hospital.

Internet use was virtually absent after the first two days of hospitalization (Figure 1). Therefore, behaviors of internet use and the outcome variables are presented in Table 2 only for the first two days of hospitalization and at discharge. Statistically significant differences within the two subgroups (with and without internet use) were observed on the UIH-VAS scale.

**Table 1:** Sample characteristics stratified by internet usage for seeking health-related information at home.

		N	Did not use the internet before hospitalization (n=7)	Used the internet before hospitalization (n=98)	All subjects	*P* value
Age (years), median (IQR)		105	35 (34-38)	33 (29-36)	33 (29-36)	.12
**Education level, n (%)**		105				.11
	University degree		3 (43)	50 (51)	53 (50.5)	
	Primary school		2 (29)	4 (4)	6 (5.7)	
	High school		2 (29)	44 (45)	46 (43.8)	
No alcohol, n (%)		105	7 (100)	92 (94)	99 (94.3)	.50
No smoking, n (%)		105	7 (100)	95 (97)	102 (97.1)	.64
**Previous pregnancies, n (%)**		105				
	0		2 (29)	50 (51)	52 (49.5)	.45
	≥1		5 (72)	48 (49)	53 (50.4)	
**Outcome of pregnancies, n (%)**		53				
	Birth at term		3 (43)	30 (31)	33 (31)	.50
	Premature		1 (14)	2 (2)	3 (3)	.06
	Stillbirth		0 (0)	1 (1)	1 (1)	.79
	Miscarriage		2 (29)	26 (27)	28 (27)	.90
	Extrauterine pregnancy		0 (0)	1 (1)	1 (1)	.79
	Hydatidiform mole		0 (0)	0 (0)	0 (0)	
**Actual pregnancy problem, n (%)**		105				
	Gestational hypertension induced, pre-eclampsia, eclampsia		0 (0)	11 (11)	11 (10.5)	.64
	Partial placental abruption		1 (14)	1 (1)	2 (1.9)	.01
	Placenta previa		0 (0)	4 (4)	4 (3.8)	.59
	Breech presentation of the fetus		0 (0)	1 (1)	1 (1.0)	.79
	Gestational diabetes		0 (0)	7 (7)	7 (6.7)	.46
	Intrahepatic cholestasis of pregnancy		0 (0)	14 (14)	14 (13.3)	.28
	Pregnancy hyperemesis		0 (0)	3 (3)	3 (2.9)	.64
	Twin pregnancy		1 (14)	15 (15)	16 (15.2)	.94
	Risk of premature birth		3 (43)	38 (39)	41 (39.0)	.83
	Intrauterine growth restriction		0 (0)	8 (8)	8 (7.6)	.43
	Other		3 (43)	26 (27)	29 (27.6)	.66
**Tool used to search health information, n (%)**		105				
	Smartphone		2 (29)	79 (81)	81 (77.1)	.002
	Tablet		0 (0)	2 (2)	2 (1.9)	.70
	Notebook		1 (14)	32 (33)	33 (31.4)	.31
	Personal computer		4 (57)	3 (3)	7 (6.7)	<.001
**Last time of internet use for health information, n (%)**		95				.85
	Within the last week		5 (83)	79 (89)	84 (88)	
	Within the last month		1 (17)	9 (10)	10 (11)	
	Within the last year		0 (0)	0 (0)	0 (0)	
	Over a year ago		0 (0)	1 (1)	1 (1)	
**Usefulness of online information about the pathology, n (%)**		95				.09
	Very useful		2 (33)	7 (8)	9 (9)	
	Somewhat useful		3 (50)	73 (82)	76 (80)	
	A little useful		1 (17)	9 (10)	10 (11)	
	Not at all useful		0 (0)	0 (0)	0 (0)	
Do not share online health information with midwife or gynecologist, n (%)		93	3 (50)	46 (53)	49 (53)	.89
**To what degree do you feel safe consulting the internet for advice or information on pregnancy?, n (%)**		95				.84
	Completely safe		0 (0)	2 (2)	2 (2)	
	Very confident		0 (0)	11 (12)	11 (12)	
	Fairly confident		5 (83)	55 (62)	60 (63)	
	Shortly confident		1 (17)	20 (22)	21 (22)	
	Not at all confident		0 (0)	1 (1)	1 (1)	
STAI^a^ trait score, median (IQR)		105	45 (42-47)	47 (45-50)	47 (44-50)	.18
EQ-5D^b^ score, median (IQR)		104	0.80 (0.57-0.85)	0.76 (0.69-0.88)	0.76 (0.69-0.88)	.96

^a^STAI: State-Trait Anxiety Inventory.

^b^EQ-5D: EuroQOL 5 dimensions.

**Table 2:** Behavior of internet use, health status, critical events, anxiety, and drug use on the first two days of hospitalization and at discharge.

		Day 1				Day 2				Discharge			
		All (N=105)	Internet use (n=81)	No internet use (n=24)	*P* value	All (N=105)	Internet use (n=57)	No internet use (n=48)	*P* value	All (N=105)	Internet use (n=11)	No internet use (n=94)	*P* value
State of health today (VAS^a^ scale), median (IQR)		59 (50-70)	50 (50-65)	70 (60-80)	<.001	60 (50-70)^b^	50 (50-70)	70 (50-70)	.004	80 (70-90)^c^	50 (50-50)	80 (70-90)	.004
**Critical events that altered your emotional state today, n (%)**													
	Family	N/A^d^	0 (0)	N/A	N/A	N/A	0 (0)	N/A	N/A	N/A	0 (0)	N/A	N/A
	Obstetric	N/A	76 (95)	N/A	N/A	N/A	54 (95)	N/A	N/A	N/A	11 (100)	N/A	N/A
	Hospital-related	N/A	3 (4)	N/A	N/A	N/A	2 (4)	N/A	N/A	N/A	0 (0)	N/A	N/A
	Other	N/A	1 (1)	N/A	N/A	N/A	(1 (2)	N/A	N/A	N/A	0 (0)	N/A	N/A
UIH^e^-VAS, median (IQR)		20 (0-25)^f^	20 (0-29)	0 (0.00-8.75)	.02	0 (0-20)	20 (0-30)	0 (0-0)	<.001	0 (0-0)	0 (0-0)	0 (0-0)	.84
STAI^g^-state score, median (IQR)		42 (41-44)	42 (41-44)	42 (40-44)	.83	42 (41-44)	42 (40-44)	42 (41-44)	.55	41 (40-43)	41 (38-43)	41 (40-44)	.11
Use of drugs, n (%)		93 (88.6)	73 (90)	20(83)	.36	86 (81.9)	52 (91)	34 (71)	.007	47 (44.8)	7 (64)	40 (43)	.18

^a^VAS: visual analog scale.

^b^N=103.

^c^N=104.

^d^N/A: not applicable; these data were only assessed among subjects that reported using the internet.

^e^UIH: Use of Internet Health-information.

^f^N=81.

^g^STAI: State-Trait Anxiety Inventory.

**Figure 2:**
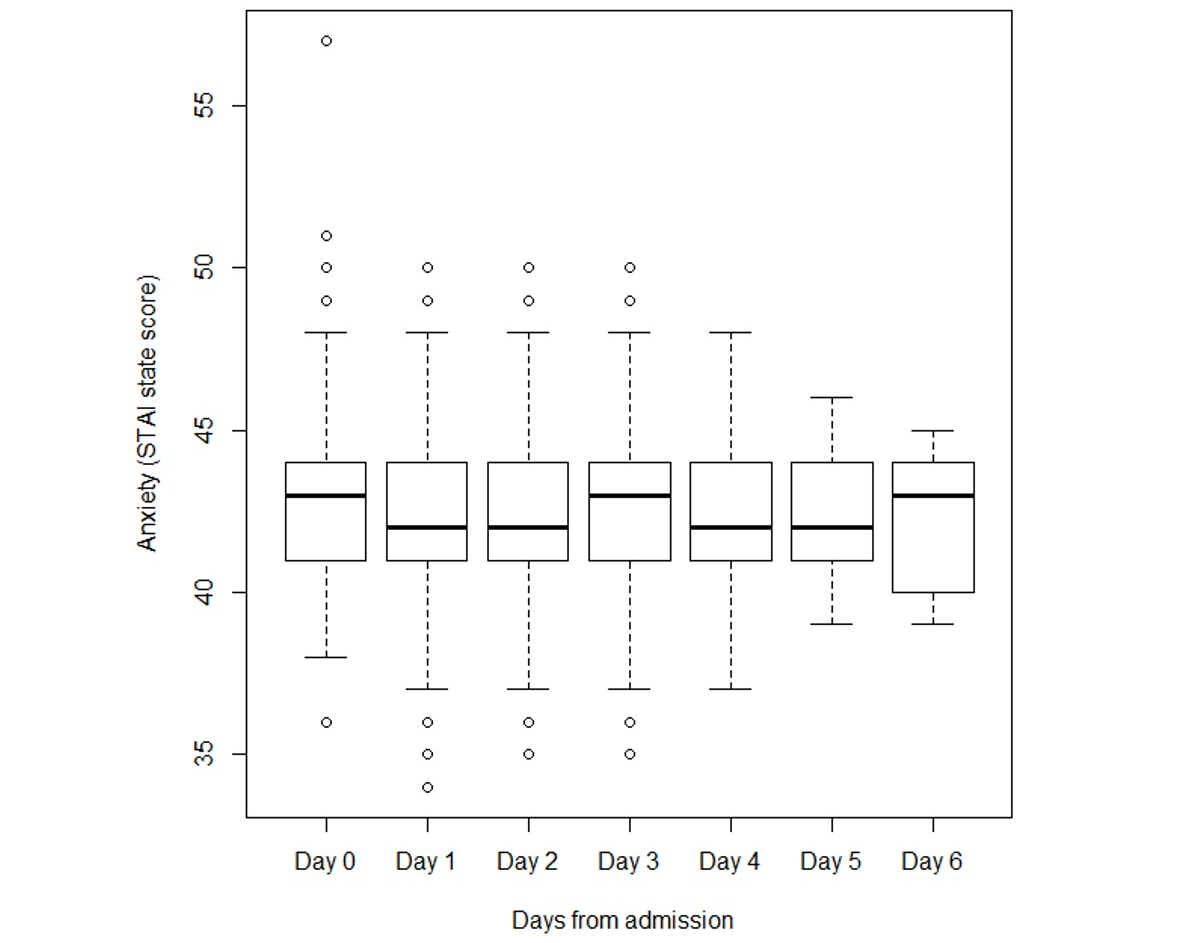
Anxiety levels, determined by the State-Trait Anxiety Inventory (STAI) state score during the first 6 days of hospitalization from admission (*P*=.33).

Anxiety levels were stable over time (Figure 2). Overall, the results indicated that using the web as a source of health information does not substantially increase anxiety levels.

**Figure 3:**
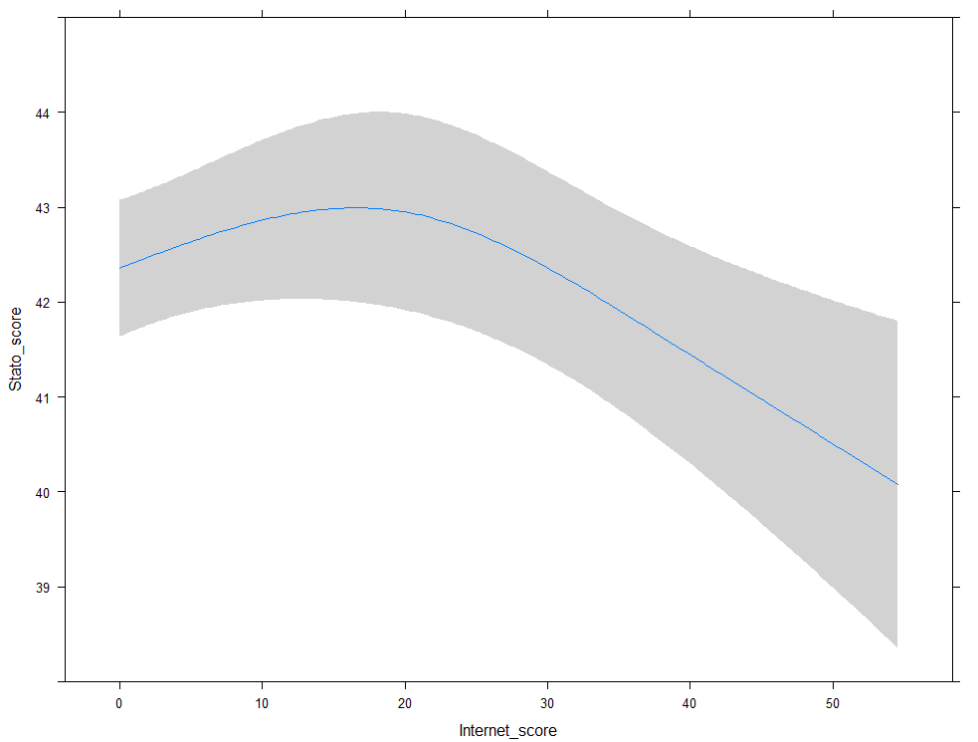
Association of UIH-VAS and STAI-State score. Non linearity (*P*=.007) estimated via restricted cubic splines and adjusted for EQ5D-VAS, age, critical events, and drug consumption. UIH: Use of Internet Health-information Questionnaire; VAS: visual analog scale; STAI: State-Trait Anxiety Inventory; EQ5D: EuroQOL 5 dimensions questionnaire.

A multivariate model was used to estimate the association between STAI scores and UIH-VAS in the first two days of hospitalization (Table 3). Only the UIH-VAS scale showed a significant nonlinear association (*P*=.007), which remained significant after adjustment for major confounding factors (Figure 3). No significant interaction was found between UIH-VAS and time (day 1, day 2) on STAI (*P=*.51).

**Table 3:** Multivariate model for State-Trait Anxiety Inventory score.

Covariate	Effect^a^	SE	Lower 0.95	Upper 0.95	*P* value
Age (7-year difference)	–0.435	0.239	–0.906	0.035	.07
EQ-5D^b^-VAS^c^ (0.20 points difference)	0.009	0.145	–0.276	0.296	.95
UIH^d^-VAS (20 points difference after 30 points)	–1.855	0.596	–3.031	–0.680	.007
Drug consumption (no vs yes)	–0.157	0.570	–1.282	0.967	.78
Critical events (occurrence vs nonoccurrence)	–0.444	0.450	–1.332	0.443	.32

^a^Effect is the slope of the linear regression model for each covariate expressed in terms of the interquartile difference for continuous covariates and using a reference category for categorical variables; for UIH-VAS, the effect is nonlinear.

^b^EQ-5D: EuroQOL 5 dimensions.

^c^VAS: visual analog scale.

^d^UIH: Use of Internet Health-information.

## Discussion

### Principal Findings and Interpretation

The results of the present study need to be interpreted in light of the related literature on network system expansion [29,30]. An increasing number of people are browsing the internet daily to obtain any type of information. Access and usage of the internet is now nearly ubiquitous, which poses new challenges for health care practitioners and users, and the terms “pregnancy” and “obstetrics” are among the top 5 searched medical keywords [31]. In addition, when defining online health communication as sending emails about health matters to family or health care providers [32], 52.4% of the women (55/105) that had internet access in our study reported sending emails or using the internet to communicate with a doctor about their health.

Although we did not find significant associations between factors such as age or education with internet use, this effect partially reflects findings from previous studies [24] as we found a large diffusion of web use among a relatively young sample (median 33 years old), with 93% of the population accessing the internet to obtain nonspecific information about health.

Studies published in the early 2000s indicated moderate use of online health information-seeking by internet users in the general population [33,34]. Conversely, but not surprisingly, despite focusing only on pregnant women in this study, we found a high percentage of women using the internet to search for information about pregnancy problems before hospital admission (82%). Other studies showed that 91% of the surveyed women had access to the internet, 84% of whom used it to search for information related to their condition, especially in the early stages of gestation, whereas 70% of these women did not talk to their health care providers about the health information they found online [35]. Since half of the information sought by the women in our sample was suggested by physicians, the internet was used most likely used to obtain information that could confirm the diagnosis or provide further details on the topic. Nevertheless, the women in our cohort also did not largely discuss what they found with physicians, probably because they felt that their health care providers would not accept the internet as a reliable source of medical information [35]. Finally, patients are usually considered as passive recipients of information rather than being treated as the main actors in their health course, as it should be. This general situation can also be applied to pregnant women who seek support and a sense of community in relation to their condition [11].

### Strengths and Limitations

To our knowledge, this is the first study to directly evaluate internet use by pregnant women during hospitalization for obstetric problems. Although hospitalization causes an increase in anxiety levels in this vulnerable population, our results showed that use of the internet to search health information reduces anxiety levels. The reason behind this finding could be related to the effect of the acquisition of information itself; that is, anxiety (state anxiety) can be reduced when pregnant women become more aware about their clinical condition (ie, the prognosis of the disease and its management). The majority of information received from the internet was obtained in the first 2 days of hospitalization. The reduction in internet usage from the third day of hospitalization is likely due to the longer time spent in contact with physicians, the influence of setting and health care providers, and clinical improvement. Consequently, the information sought during the first few days of hospitalization likely helped the pregnant women in reducing their anxiety levels.

Moreover, pregnant women often receive limited basic information on prenatal health behaviors. Patients often perceive this information to be inconsistent and inadequate, which could also explain why they search for information online and do not share it with their physicians [36]. This suggests that current assistance approaches to pregnancy may not fully respond to patients’ information demand. This might be due to the limited time of direct contact between patients and their health care providers but also due to the unpredictability of the onset of disease and the complexity of diagnosis. Moreover, disease management by midwives requires time for both communicating information and understanding it.

Pregnancy disorders have a clear impact on the perception of anxiety; consequently, the risk of adverse events for the mother and her baby imposes some lifestyle changes to a pregnant woman. In this context, information can play an important role on women’s psychological status: improved knowledge about a disease will increase a patient’s perceived self-efficacy and the ability to develop adaptive coping strategies. The internet offers the possibility to remain connected with the virtual community of pregnant women and physicians and to obtain all types of information, making internet users more confident in how to manage their condition [32,37]. Some authors also hypothesized that people looking for medical advice and health information are more predisposed to pay greater attention to and be more interested in their clinical condition, resulting in a higher self-efficacy perception [33,36].

Interestingly, our study showed a quite substantial potential impact of the internet in reducing anxiety. Patients with higher internet usage behavior reported an anxiety level that was 2 points lower than that of patients with less intense internet usage (42 vs 40 points, *P*=.008). This effect accounts for approximately one quarter of the effect of more aggressive therapies in reducing pathological anxiety, such as serotonin reuptake inhibitors combined with psychotherapy, and psychotherapy treatment alone, and accounts for approximately one half of the effect of cognitive behavioral therapy and other unconventional therapies [24-39]. Since anxiety is modulated by many intersectional factors, it might be interesting to further evaluate the effect of internet usage in association with other types of treatments for anxiety in pregnant women, even if a diagnosis of pathological anxiety would be necessary and certain antianxiety drugs cannot be administered to pregnant women.

This study also has several limitations. First, the UIH questionnaire, despite being validated, is not very detailed in terms of assessing the quality of internet usage. Furthermore, data on the specific websites visited would have provided a more precise framework of internet usage. This might be particularly important in assessing the quality of the obtained information about the disease and its subsequent impact on anxiety and other forms of psychological distress. Moreover, the names and types of websites would have been useful to investigate the emotional status in relation to active (eg, sharing health information with others) and passive (eg, simple search for information for personal purposes) use of the internet.

Finally, because of the nature of this study, causal interpretation of the association between exposure to the internet and the level of anxiety is not possible, making the potential interpretation on the “therapeutic” psychological effect of internet usage merely speculative at this point.

### Conclusions

This study has implications for health care providers, suggesting that the internet could offer a useful instrument to support clinical practice due to its informational power and its potential impact on well being–related outcomes. The widespread search for online health information among women with pregnancy-related diseases mainly focuses on the possible outcomes for the baby and on the quality of communication between patients and health care providers, emphasizing the role of the internet as a potential tool for enhancement of such essential communication.

To effectively influence the online experiences of pregnant women, professionals involved in the childbirth pathway should have a basic understanding of the internet and learn how to actively engage women’s interest in the internet. For this purpose, the installation of free wifi areas in maternity departments could be useful.

## Abbreviations

EQ-5D: European quality of life 5 dimensions questionnaire
STAI: State-Trait Anxiety Inventory
UIH: Use of Internet Health-information Questionnaire
VAS: visual analogic scale
